# Anti-Inflammatory State in Arabian Horses Introduced to the Endurance Training

**DOI:** 10.3390/ani9090616

**Published:** 2019-08-27

**Authors:** Olga Witkowska-Piłaszewicz, Piotr Bąska, Michał Czopowicz, Magdalena Żmigrodzka, Ewa Szarska, Jarosław Szczepaniak, Zuzanna Nowak, Anna Winnicka, Anna Cywińska

**Affiliations:** 1 Department of Pathology and Veterinary Diagnostics, Faculty of Veterinary Medicine, Warsaw University of Life Sciences, 02-787 Warsaw, Poland; 2 Division of Pharmacology and Toxicology, Department of Preclinical Sciences, Faculty of Veterinary Medicine, Warsaw University of Life Sciences, 02-787 Warsaw, Poland; 3 Laboratory of Veterinary Epidemiology and Economics, Faculty of Veterinary Medicine, Warsaw University of Life Sciences, 02-787 Warsaw, Poland; 4 Military Institute of Hygiene and Epidemiology, 01-163 Warsaw, Poland; 5 Department of Animal Nutrition and Biotechnology, Faculty of Animal Sciences, Warsaw University of Life Sciences, 02-787 Warsaw, Poland; 6 Department of Genetics and Animal Breeding, Faculty of Animal Sciences, Warsaw University of Life Sciences, 02-787 Warsaw, Poland

**Keywords:** cytokine, inflammatory response, sport, equine, comparative immunology

## Abstract

**Simple Summary:**

The aim of this study was to investigate whether endurance training changes pro- and anti-inflammatory cytokine profile within a 20-week training season in young inexperienced endurance Arabian horses. It has been suggested that regular endurance training can induce an advanced anti-inflammatory response, but its nature is not completely understood. However, to promote more effective use of endurance exercise training in health promotion and disease prevention, a complete understanding of the nature of its immune regulatory effect is required. The results suggest that endurance training can induce advanced overall anti-inflammatory response as an adaptation to increasing workload.

**Abstract:**

Development of an anti-inflammatory state during physical training has been postulated in both human and equine athletes, but it is not completely understood. The aim of this study was to investigate whether endurance training changes pro- and anti-inflammatory cytokine profiles within a 20-week training season in young inexperienced endurance horses. Nine Arabian horses were examined in this prospective 20-week follow-up study. Blood samples were analysed 5 times monthly, at rest and after training sessions. Routine haematological examinations were performed. Cytokine patterns including IL-1β, IL-6, TNF-α, IL-10 mRNA expression using Real Time-PCR, and serum concentrations of IL-1β, IL-2, IL-4, IL-6, IL-17, INFγ, TNF-α, and IL-10 by ELISA test were determined. During endurance training, the most significant decrease in post-exercise cytokine type 1 levels (TNFα and IL-β) occurred within 20 weeks, beginning from the 3rd month of training. IL-6 serum level decreased after the 4th month. The results suggest that endurance training can induce advanced overall anti-inflammatory response as an adaptation to increasing workload.

## 1. Introduction

Horses and humans share unique features that allow them to be involved in competitive sport. Success in elite sporting events requires natural athletic capacity and regular long-lasting training. Although both the metabolism and training programs differ greatly between horses and humans, the regular physical effort results in many adaptational changes, and for some of them, including the immunological ones reflecting health status, horses can be considered a reference species for humans [1,2]. Horses seem particularly useful for these studies, as in contrast to humans, they are not involved in other activities like professional work, which can disturb the training program and cause additional stress, so long-lasting training in the horses can be monitored more precisely. 

There are several similarities between ultra-endurance human exercise, such as an Ironman triathlon or ultra-marathon, and equine endurance rides at the longest distances. These activities are extraordinary in the level of strenuous exercise performed; however, substantial differences exist among endurance disciplines and between the training regime in humans and horses. In horses, the appropriate workload during training is much lower than in humans, and levels comparable to those undertaken by humans would almost systematically result in lameness [3]. In young endurance horses, the training begins with a long slow distance method, which is also recommended with the appropriate workload for undertrained or moderately trained humans. Ultra-marathoners run much slower than marathoners [4], but complete more running kilometres and more running hours per week in training. The key predictors of a successful ultra-marathon finish are age (30–50 years for men and 30–55 years for women) and specific aspects of anthropometry including low body fat and low body mass index (BMI) [5]. Equine endurance sports also require special anatomical and physiological predispositions, and these criteria are fulfilled by Arabian horses [6,7]. Due to certain limitations in the studies on the effects of ultra-endurance exercise, especially at the beginning of training, endurance horses, particularly those that have not previously been involved in any performance activity, may be considered a good model for human athletes, at least in some aspects, including the adaptation of the immune system. 

Exercise-induced changes in the blood concentrations of certain indicators of acute phase response (APR), the nonspecific systemic reaction induced by any kind of disturbances in homeostasis, have been studied in humans for years, and in the last decade also in horses [8,9,10]. It has been shown in both ultramarathoners and elite endurance horses that the blood concentrations of some parameters change in an APR-like pattern, while others remain unchanged [11,12]. In humans, pro-inflammatory cytokines (e.g., interleukin 1β-IL-1β, TNF-α—tumour necrosis factor α), which are involved in APR, temporarily increased during and shortly after prolonged exercise [13]. The precise mechanisms mediating the exercise-induced APR have not been clarified; however, the increases in various anti-inflammatory mediators such as IL-1 receptor antagonist (IL-1ra) or interleukin 10 (IL-10) during longitudinal training have been shown [14]. Moreover, it has been postulated that long-lasting training results in decreased APR after strenuous activity as an adaptation to increasing workload during training [15,16]. Several mechanisms responsible for anti-inflammatory effects have been proposed, including the release from working skeletal muscles of interleukin-6 (IL-6) and other myokines, as well as subsequent increases in circulating levels of IL-10 and IL-1ra; increased circulating numbers of IL-10-secreting regulatory T cells or decreased number of circulating monocytes; and downregulation the Toll-like receptor expression [17]. However, most of the published results apply to acute exercise and short-lasting observations (up to 9 weeks) [15]. Many studies compare the immune responses following various types of physical activity in either trained [18,19] or untrained individuals [20], but much less is known about the changes in immune mechanisms resulting from prolonged exercise, regularly repeated during the training process. Some observations indicate that regular and systematic physical training can lead to create an anti-inflammatory, protective state [17,21,22,23], although the development of such a condition is still poorly understood. 

Thus, the aim of this study was to investigate the changes of cytokine profile, reflecting pro- and anti-inflammatory response in the long period during the first season of endurance training in Arabian horses.

## 2. Materials and Methods 

### 2.1. Animals

Nine privately owned, healthy 6–7-year-old Arabians (2 mares and 7 geldings) were enrolled in this study. The horses had not undergone any regular training previously. They were kept in two endurance training centres, and were fed and trained according to the same protocols in similar terrain; no changes to the normal routine were made due to this study. The horses were monitored for 5 months (20 weeks) following their first training season. All exercises were provided under similar terrain conditions. The training involved daily sessions with exercise load depending on horse condition and increasing with time; altogether, the horses covered about 250 km per month, and every 14–20 days, sessions with high exercise load were performed. Beginning with the 3rd month of training, the horses were introduced to endurance competitions over limited distance with limited speed (42 km, speed 10–16 km/h). The animals were examined before and after training sessions selected for the study—the ones performed every 14–20 days, with high exercise load (25–28 km, covered with the speed 14–15 km/h). Clinical examinations were performed in the morning and after training and revealed no clinical abnormalities during the whole observation period. The examination mirrored the standard clinical examination at vet gates during competitions and included heart rate, mucous membranes (colour and moisture), dehydration (measured as the time it takes for a pinched skin fold over the point of the shoulder to flatten), gut sounds, muscle condition and regularity of gait (evaluated in trot). Five monthly training sessions of maximal load, the ones every 14–20 days, mentioned above, were included in the analysis. Due to the fact that the horses did not exercise together, the number of horses in each training session varied. 

### 2.2. Sampling

Blood samples were taken at rest, 8 a.m. (2 h after feeding), and after training (about 2.5 h after the rest measurement), as a part of standard veterinary diagnostic procedures. Therefore, no approval of the Local Commission for Ethics in Animal Experiments was required, according to the Polish legal regulations [24] and the European directive EU/2010/6. All samples were acquired by jugular venepuncture using BD Vaccutainer system into: K2-ethylenediaminetetraacetic acid (K2-EDTA) tubes for haematological tests, dry tubes for serum analyses and Tempus Blood RNA Tubes (Applied Biosystems) for analyses of cytokine mRNA expression. EDTA blood samples were kept at +4 °C and examined within 5 h for the following haematological parameters: white blood cell count (WBC), packed cell volume (PCV), haemoglobin concentration (HGB), red blood cell count (RBC) and platelet count (PLT) using an automated analyser calibrated for equine species (ABC Vet, Horiba ABX). 

Dry tubes were centrifuged (4380× *g*, 5 min) and serum was aspirated for further analyses (biochemistry and ELISA tests). Clinical biochemistry analyses including aspartate aminotransferase (AST) and creatine phosphokinase (CPK) activity were performed with automated clinical biochemistry analyser (Miura One, ISE. S.r.l., Rome, Italy). Total protein concentration (TP) was measured by refractometer technique (Reichert Rhino Vet 360). For all measurements, Pointe Scientific (USA) reagents, standards, calibrators and controls were used.

### 2.3. ELISA Test

The concentration of cytokines: IL-1β, interleukin 2 (IL-2), interleukin 4 (IL-4), IL-6, IL-10, interleukin 17 (IL-17), interferon γ (INFγ) and (TNF-α) was determined by commercially available immunoenzymatic commercial assay dedicated for equine species (Cloud-Clone Corp., Katy, TX, USA). The absorbance was measured by Multiscan Reader (Labsystem, Helsinki, Finland) using a Genesis V 3.00 software program.

### 2.4. Gene Expression Analysis 

Analyses of cytokine mRNA expression (IL-1β, IL-6, IL-10, TNF-α) were determined using RealTime-PCR method. Total RNA was extracted using the MagMax Total RNA Isolation Kit (Thermo Fisher Scientific, Waltham, MA, USA) according to the manufacturer’s instructions. RNA concentration was determined using Nano-Drop 2000 Spectrophotometer (Thermo Fisher Scientific, Waltham, MA, USA). mRNA expression was determined by quantitative PCR (qPCR) using TaqMan Master Mix (Thermo Fisher Scientific, USA) and ABI 7500 qPCR system (Life Technologies, Carlsbad, CA, USA). Gene expression was determined using the relative quantitation method [25] with β-glucuronidase (β-Gus) as the reference gene [26]. Samples were assayed using previously published sequences of primers and probes [27].

### 2.5. Statistical Analysis 

Numerical variables are presented as the arithmetic mean ± standard deviation (SD). These were considered normally distributed when SD was less than one-half the arithmetic mean of non-negative variables [28] and the Shapiro-Wilk W test was insignificant. As both criteria were satisfied in the vast majority of cases, untransformed variables were statistically analysed. The mixed linear models were developed to assess the influence of repeated trainings and the moment of blood collection on the concentration of cytokines and transcripts (Y). The moment of blood collection was included as a category “after” (X_after_) with “before” being a reference category. Subsequent trainings were included as four categories: 2nd training (X_2nd training_), 3rd training (X_3rd training_), 4th training (X_4th training_), 5th training (X_5th training_) with the 1st training acting as a reference category. Both the aforementioned explanatory variables were fitted as fixed effects. A variable “horse” was fitted as a random effect and forced into each model to control for the dependence of observations coming from a single horse in which cytokine and transcript concentrations were determined different number of times.

Y = B_0_ + B_after_ × X_after_ + B_ith training_ × X_ith training_ + H (1)

B_0_ was an intercept and B with a relevant subscript stood for the coefficient of regression of a given explanatory variable, and *H* was the random effect of a horse. All statistical tests were two-tailed, and the significance level (α) was set at 0.1 for the overall F test in the mixed linear models and at 0.05 elsewhere. Univariable statistical analysis was performed and the graphs were prepared in TIBCO Statistica 13.3.0 (TIBCO Software Inc., Palo Alto, CA, USA). Mixed models were developed in IBM SPSS Statistics 24. Given the small size of the effect expected and small sample size, the power of mixed linear models is likely to be low [29]. Thus, insignificant results should be interpreted cautiously.

## 3. Results

**Figure 1:**
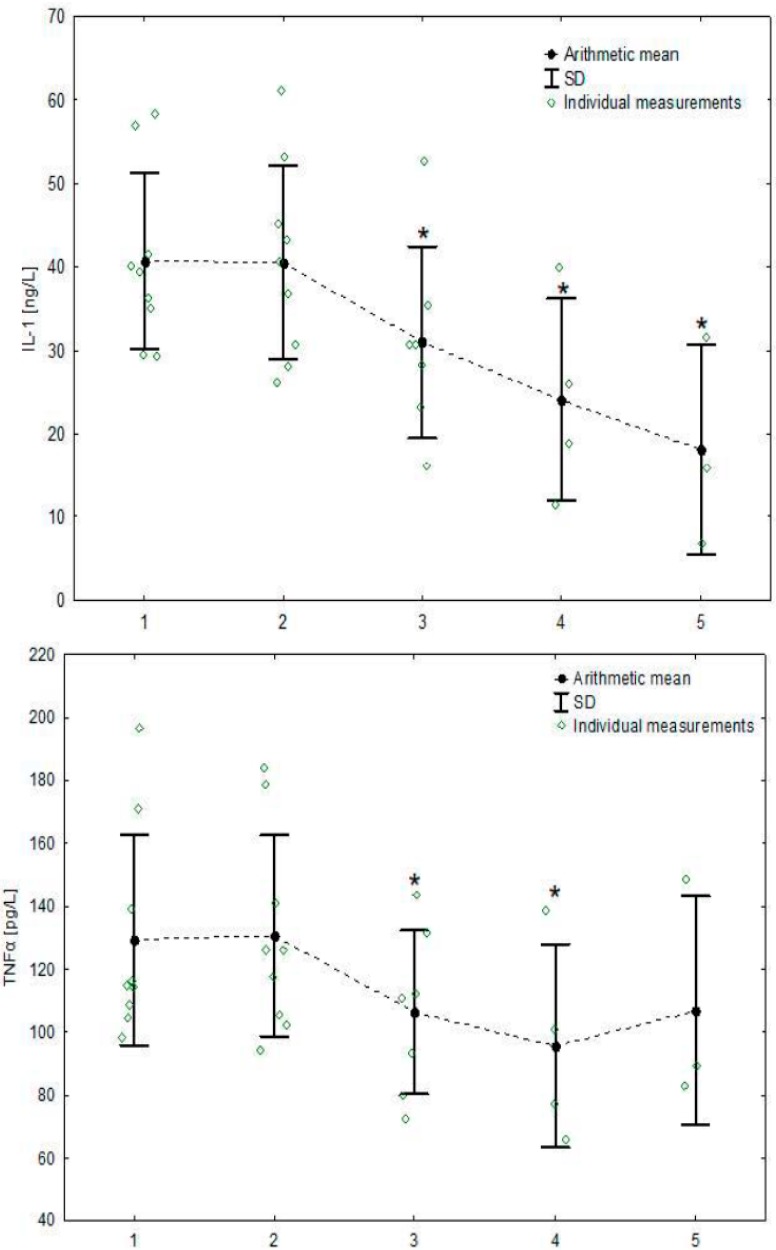

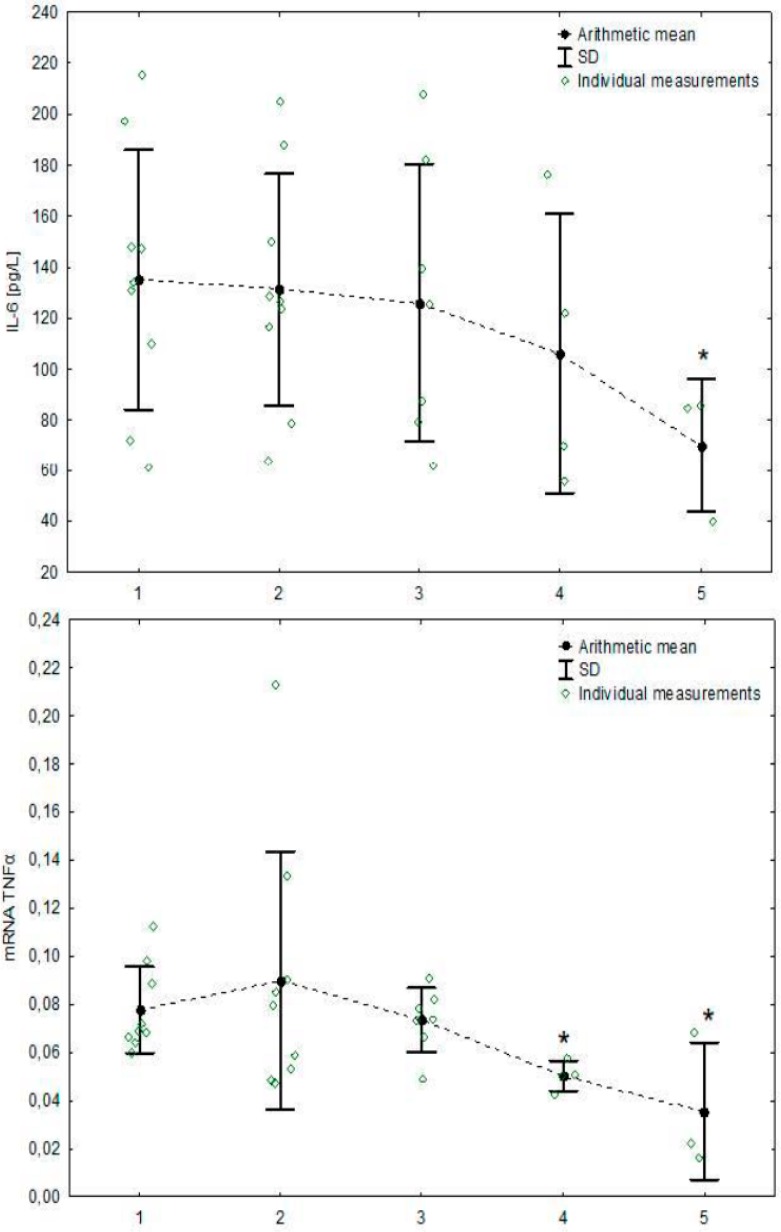

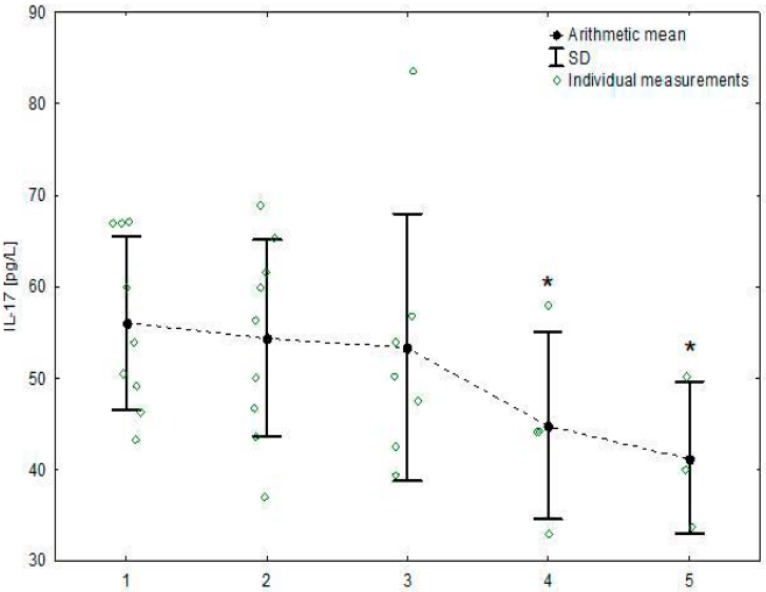
Serum concentrations of cytokines and cytokines’ mRNA expressions whose concentration changed significantly during the 5-month training season. Individual averaged measurements from before and after exercise are presented for each horse together with the mean and SD for each effort. Asterisks signify trainings with significantly lower cytokine or transcript concentration compared to the 1st training, *p* < 0.05; abbreviations: IL-1, interleukin 1; IL-6, interleukin 6; IL-17, interleukin 17; TNF-α, tumour necrosis factor α; mRNA TNFα, mRNA tumour necrosis factor α; SD, standard deviation.

All haematological and blood biochemical parameters determined before the training sessions varied within normal ranges for equine species [30]. The values measured before and after training sessions during the season are presented in Table 1 and detailed concentrations of cytokines in Table 2. Neither serum cytokine concentrations nor cytokine mRNA expression changed significantly after training sessions. However, significant decreases from the initial values were observed (Table 3) in the concentrations of 4 cytokines: IL-1*β* (F_4,51_ = 13.28, *p* < 0.001), IL-6 (F_4,50_ = 2.29, *p* = 0.072), IL-17 (F_4,51_ = 2.07, *p* = 0.098) and TNF*α* (F_4,51_ = 10.08, *p* < 0.001) and mRNA TNF*α* transcript (F_4,52_ = 3.58, *p* = 0.012). Concentrations of IL-1*β* and TNFα started to decrease after the 2nd training, concentration of IL-17 after the 3rd training, and concentration of IL-6 and expression of mRNA TNFα after the 4th training (Figure 1). The concentrations of the remaining cytokines and the expression of transcripts remained unchanged (Tables S1 and S2).

## 4. Discussion

This is the first study presenting the changes in blood cytokine concentrations in endurance horses monitored for 20 weeks of their first training season. We intended to examine the training under field, rather than experimental, conditions; thus, the main limitation was the low number of horses, which decreased with time. As in all field studies, we had to respect the training regimen, the owners’ plans, etc.; thus, sometimes one part of examined group were being trained at one centre and the other part at the second centre at the same time, so it was possible to examine only one part. Due to these limitations, our results should be considered as preliminary; however, an interesting tendency is clearly visible. Observed decreases in cytokine levels confirm the reduction of inflammatory capacity and possibly further induction of an anti-inflammatory condition by longitudinal endurance training. Previous studies have demonstrated a similar phenomenon, indicated by an overall reduction in the expression of proinflammatory cytokines in Thoroughbred horses undergoing race training [23]. In humans, too, a similar condition has been suggested on the basis of increased production and release of anti-inflammatory cytokines from contracting skeletal muscles [17]. 

Our results were analysed in the context of haematological and blood biochemical parameters, commonly accepted in the monitoring of endurance training. Regular monitoring of haematological measurements is useful for the evaluation of training progress in individual horses; however, it has little value for assessing the fitness of the group due to large variations among animals [31,32,33]. Similarly, in humans, there are still no tests that work well enough at both individual and group levels [34]. In our study, we noticed typical increases in haematological parameters after training sessions; however, they were not significant at the group level. 

The local inflammatory process is considered fundamental for inducing muscle remodelling in response to exercise [35,36]. In humans, only limited data regarding changes in the post-exercise level of pro-inflammatory cytokines such as IL-1β, IL-2, IL-6, IL-8, INFγ and TNF-α after ultra-endurance exercise are available [37,38]. These studies indicated that in highly trained men, the blood concentrations of IL-1β, INFγ and TNF-α remained unchanged after ultra-endurance exercise (triathlon), but IL-6, IL-10 and IL-1ra markedly increased [38]. The same pattern has been identified in elite endurance horses after 120-km and 160-km endurance rides, suggesting the presence of anti-inflammatory state in the horses ready for competing at the longest distances [39]. Taking into account the sampling time, it was concluded that in these horses, type 1 cytokines promoted the development of exercise-induced acute phase response which did not result in inflammation due to inhibition by anti-inflammatory IL-10 [39]. Pro-inflammatory cytokines are divided into two major groups: IL-1-type cytokines (including IL-1 and TNF-α), which elicit a primary autostimulatory signal, stimulating the release of a secondary cytokine signal; and IL-6-type cytokines (including IL-6), which may exert a negative feed-back on the production of IL-1-type cytokines [40]. 

In our study, the concentrations of all examined pro-inflammatory cytokines (IL-1*β*, IL-2, IL-6, IL-17, INFγ and TNF-*α*) in blood after each training session remained unchanged at group level when compared to pre-exercise values, and mean values were taken for the analysis of long lasting effects. These effects were clearly seen as the reduction of the concentrations of type 1 cytokines (IL-1*β* and TNF-*α*) beginning from the 3rd training, which seems to indicate that the development reduced inflammatory capacity. Further reduction of inflammatory capacity was seen in subsequent trainings, including a decrease in IL-6 level at the 5th one. IL-1 and TNF α upregulate IL-6 production [41]; thus, the decreases that occur from the 3rd training may have also led to the decrease in IL-6 at the 5th one. Although increases in anti-inflammatory cytokine levels did not occur in our study, it is likely that this progressive reduction of inflammatory capacity could also further result in an overall “anti-inflammatory state”, as seen in elite competing horses [39]. 

The plasma concentration of IL-6 in response to strenuous exercise increases more than any other cytokine [42]. It has been demonstrated that IL-6 is involved in the control of the early inflammatory response to physical exercise in both humans and equids [39,43]. Recently, the release of IL-6 by muscles has been considered an important factor regulating metabolism and stimulating the regenerative and proliferative processes of the satellite cells [44]. Importantly, some studies have suggested that IL-6 levels remains significantly above pre-race levels until the 5th day post-race in long-distance triathlons [45] and intense interval training in triathletes [46]. In our study, no elevations of IL-6 were noted after endurance training sessions. Thus, the workload during single training session was not high enough to produce muscle injury and IL-6 secretion, observed after long distance rides [39]. It was documented that the increase of IL-6 depends on exercise intensity, duration, the mass of muscle recruited, and endurance capacity of the athlete [43]. In our study, single training had no impact on IL-6 level, but the cumulative effect of the training process has been seen.

IL-6 can have both pro- and anti-inflammatory action [47]. In our study, the IL-6 serum level significantly decreased in the 5th month of training. We hypothesized that the higher level of IL-6 at the beginning of training season might prevent the production of type 1 pro-inflammatory cytokines. This kind of IL-6 action has been confirmed in other experiments. IL-6 has been shown to inhibit lipopolysaccharide (LPS)-induced IL-1 and TNF-α production in human monocytes and in the human monocytic line U937 [48]. Moreover, IL-6 infusion, as well as exercise, inhibited the endotoxin-induced increase in circulating levels of TNF-α in healthy humans [49]. Furthermore, an increase in IL-6 during strenuous exercise triggers the release of cytokine inhibitors, such as IL-1ra, soluble tumour necrosis factor receptor 1 (sTNF-r1), and soluble tumour necrosis factor receptor 2 (sTNF-r2), the anti-inflammatory cytokine IL-10 and pleiotropic cytokine IL-4 [14,50,51]. In our study, IL-4 and IL-10 level remained unchanged during the training season. IL-4 anti-inflammatory action involves suppression of LPS-induced TNF-α and IL-1 production [52] and induction of the synthesis of the IL-1 receptor antagonist in human monocytes [53]. Moreover, recent studies indicate that IL-4 also promotes myoblast recruitment, fusion and growth [54]. 

The results presented in human studies regarding the development of anti-inflammatory state are sometimes conflicting, or at least incompatible, which is not surprising due to various length of studies. Most of them cover up to 9 weeks of physical training [15,55,56,57]. Our results confirmed that the development of anti-inflammatory state during endurance training is a time-consuming process and in horses begins after 2 months of regular training.

Trainers’ observations and the results of the competitions have indicated that horses better tolerate the workload at the end of the 5-month study than at the beginning of training season. It is to be expected that this will be accompanied by muscle remodelling, and it has been proven that the immune system adapts, as well. This immune adaptation is likely to balance the stimulation by increasing workload during training and so that inflammation does not develop in the muscles. 

The mechanism leading to anti-inflammatory state during training is unknown. In humans, the anti-inflammatory condition has been indicated primarily on the basis of the basal level of acute phase proteins (APPs), mainly CRP—a major APP in human, but not cytokines. The level of APPs has only been compared between well-trained human athletes/sport students and untrained individuals, but changes during long-lasting training have not been analysed [15,16,58]. It has even been found surprising that CRP decreased with training, as physical exercise had been expected to be associated with an inflammatory reaction of muscles and tendons [16]. On the other hand, the production of APPs directly results from cytokine stimulation. Rapid APPs synthesis, e.g., CRP and serum amyloid A (SAA—a major APP in horses [59]), is induced by type 1 cytokines, whereas the IL-6 type-dependent pathway promotes the synthesis of the second-line APPs, like haptoglobin [40]. 

In our study, we did not observe any post-exercise changes in either pro- or anti-inflammatory cytokines’ gene expression. In experienced marathoners, a 3-h run has been shown to promote the increases in mRNA for IL-1β, IL-6, IL-8, TNFα in skeletal muscles [60], yet the systemic changes were relatively minor. Also, after prolonged exercise (a marathon), IL-6 mRNA has been found to be expressed mostly locally in the skeletal muscles and blood mononuclear cells were only an additional source [61]. Moreover, it has been postulated that sometimes the changes in some blood cytokine concentrations and their mRNA expression might be too transient to be detected [61].

In humans, the expression and degradation of mRNA is regulated transcriptionally and post-transcriptionally by several proteins and microRNAs (miRNA) [62]. Activation of these proteins and miRNAs determine the fate of any mRNA, including the cytokines’ mRNA. Moreover, it has been postulated that mRNA expression might be useless in predicting protein expression levels [63]. In our study, we noticed only a decrease in TNF-*α* mRNA expression with simultaneous decrease of post-exercise TNF-*α* in blood. It seems that in exercise, the cytokine concentrations indicate the athlete status better than mRNA expression in blood cells. 

## 5. Conclusions

Our results indicate that high load endurance training in horses leads to the development of reduced inflammatory capacity, confirmed by a decreased pro-inflammatory type 1 cytokine concentrations over time and possibly further anti-inflammatory state. The first three months of training appear to be crucial in training adaptation, the significant anti-inflammatory changes occur between the 2nd and 3rd month, and the cumulative effect of the training process can be considered. It is also presumed that IL-6 can mediate the protective, long-term anti-inflammatory effects of exercise by orchestrating an anti-inflammatory reaction. 

## Figures and Tables

**Table 1:** Haematological and biochemical parameters during training season in Arabian endurance horses (arithmetic mean ± SD).

	Month of the Training Season									
	1st (*n* = 9)		2nd (*n* = 9)		3rd (*n* = 7)		4th (*n* = 4)		5th (*n* = 3)	
Parameter	Before	After	Before	After	Before	After	Before	After	Before	After
WBC [10^9^/L]	6.7 ± 1.6	8.8 ± 2.3	6.9 ± 0.9	8.1 ± 3.1	6.5 ± 0.9	9.9 ± 1.8	7.4 ± 1.5	8.2 ± 1.7	7.1 ± 0.3	9.9 ± 3.1
RBC [10^12^/L]	8.4 ± 1.1	9.1 ± 1.1	8.7 ± 1.1	9.1 ± 1.0	8.8 ± 1.2	9.4 ± 0.8	8.2 ± 1.0	8.5 ± 1.1	8.2 ± 1.3	9.3 ± 2.0
HGB ^a^ [mmol/L]	8.0 ± 1.1	8.7 ± 1.1	8.0 ± 1.2	8.3 ± 0.7	7.3 ± 0.9	8.1 ± 0.7	7.5 ± 1.0	7.9 ± 1.3	7.4 ± 1.0	8.3 ± 1.7
PCV [l/l]	37.1 ± 5.5	40.4 ± 5.6	38.6 ± 5.5	40.4 ± 4.7	39.6 ± 5.5	42.4 ± 3.2	37.3 ± 4.7	38.8 ± 5.4	37.0 ± 6.3	42.1 ± 9.8
PLT [10^9^/L]	251 ± 151	367 ± 271	480 ± 235	580 ± 315	200 ± 104	316 ± 167	341 ± 187	505 ± 305	389 ± 443	432 ± 296
TP [g/L]	62 ± 5	66 ± 5	62 ± 4	66 ± 5	66 ± 3	69 ± 4	66 ± 5	70 ± 1	65 ± 3	70 ± 5
AST [U/L]	274 ± 23	300 ± 30	273 ± 32	287 ± 26	274 ± 28	301 ± 39	279 ± 58	295 ± 34	300 ± 49	351 ± 40
CPK [U/L]	318 ± 97	415 ± 189	239 ± 106	331 ± 125	366 ± 142	500 ± 180	331 ± 111	386 ± 119	320 ± 31	594 ± 419

^a^ to convert mmol/L to g/dL, multiply by 1.611; abbreviations: SD, standard deviation; WBC, white blood cell count; RBC, red blood cell count; HGB, haemoglobin concentration; PCV, packed cell volume; PLT, platelet count; TP, total protein concentration; AST, Aspartate aminotransferase; CPK, creatine phosphokinase; n—the number of horses.

**Table 2:** Concentration (arithmetic mean ± SD) of cytokines in blood samples collected before and after subsequent trainings.

	Month of the Training Season									
	1st (*n* = 9)		2nd (*n* = 9)		3rd (*n* = 7)		4th (*n* = 4)		5th (*n* = 3)	
Parameter	Before	After	Before	After	Before	After	Before	After	Before	After
IL-1β [ng/L]	40.7 ± 10.5	40.7 ± 11	41.3 ± 13.8	39.9 ± 11.8	29.8 ± 11.2	32.2 ± 12.2	21.3 ± 15.1	26.8 ± 13.3	18.7 ± 12.3	17.5 ± 13
IL-2 [pg/L]	26.1 ± 7.6	25.1 ± 7	25.7 ± 11.7	22.5 ± 6.0	29.5 ± 8.7	28 ± 8.2	24.2 ± 6.8	22.8 ± 9.1	20.3 ± 5.2	23.0 ± 6.0
IL-4 [pg/L]	36.1 ± 13.1	37.1 ± 9.3	34.3 ± 11.7	33.8 ± 7.6	32.7 ± 8.8	32.6 ± 8.0	31.3 ± 7.4	27.1 ± 6.6	30.7 ± 5.4	26.0 ± 5.8
IL-6 [pg/L]	139.6 ± 54.4	130.5 ± 50.6	132.5 ± 52.3	130.0 ± 41.6	133.3 ± 62.1	118.8 ± 47.6	109.1 ± 57.0	102.9 ± 53.6	68.4 ± 26.9	71.6 ± 25.6
IL-10 [pg/L]	236.6 ± 71.7	226.3 ± 55.3	240.6 ± 45.9	227.2 ± 49.9	241.5 ± 54.6	235.3 ± 78.1	229 ± 68.9	211.3 ± 41.7	182.8 ± 5.0	154.0 ± 48.4
IL-17 [pg/L]	57.8 ± 10.3	54.3 ± 10.8	56.2 ± 11.2	52.6 ± 10.9	50.7 ± 13.9	56.1 ± 16.6	47.6 ± 10.5	42.1 ± 10.3	45.9 ± 7.2	36.7 ± 9.6
TNFα [pg/L]	135.9 ± 34.7	122.8 ± 33.5	134.2 ± 36.6	127.2 ± 28.9	106.8 ± 26.1	106.1 ± 27.8	102 ± 28.3	89.7 ± 36.3	108.8 ± 27.9	105.3 ± 45.9
INFγ [pg/L]	41.6 ± 10.4	39.4 ± 8.6	42.2 ± 6.5	37.6 ± 9.0	38.4 ± 13.8	38.6 ± 11.0	33.2 ± 7.4	32.4 ± 6.2	33.0 ± 12.0	31.3 ± 8.4

Abbreviations: SD, standard deviation; IL-1β, interleukin 1β; IL-2, interleukin 2; IL-4, interleukin 4; IL-6, interleukin 6; IL-10, interleukin 10; IL-17, interleukin 17; TNF-α, tumour necrosis factor α; INFγ, Interferon γ; n—the number of horses.

**Table 3:** Cytokines and transcripts whose concentrations were significantly affected by repeated training in the mixed linear model.

Variable	IL-1 β			IL-6			IL-17			TNFα			mRNA-TNFα		
	Estimate of the Model ^a^	Parameter Statistics	*p*-Value	Estimate of the Model ^a^	Parameter Statistics	*p*-Value	Estimate of the Model ^a^	Parameter Statistics	*p*-Value	Estimate of the Model ^a^	Parameter Statistics	*p*-Value	Estimate of the Model ^a^	Parameter Statistics	*p*-Value
Intercept	40.38 ± 3.76	-	-	138.51 ± 15.97	-	-	57.24 ± 3.74	-	-	133.20 ± 10.10	-	-	0.09 ± 0.01	-	-
**Variables fitted as fixed effects**															
Time of blood collection															
Before	0	-	-	0	-	-	0	-	-	0	-	-	0	-	-
After	0.70 ± 1.62	0.43	0.668	−6.91 ± 4.00	−1.73	0.090	−2.40 ± 1.83	−1.31	0.196	−7.67 ± 4.25	−1.80	0.077	−0.01 ± 0.01	−2.01	0.050
Training															
1st ^b^	0	-	-	0	-	-	0	-	-	0	-	-	0	-	-
2nd	−0.15 ± 2.15	−0.07	0.943	−3.80 ± 5.33	−0.71	0.479	−1.65 ± 2.44	−0.68	0.503	−1.33 ± 5.67	0.23	0.815	0.01 ± 0.01	1.29	0.203
3rd	−10.65 ± 2.37	−4.50	<0.001 *	−6.26 ± 5.87	−1.07	0.291	−2.05 ± 2.68	−0.77	0.446	−27.00 ± 6.23	−4.33	<0.001 *	−0.01 ± 0.01	−0.67	0.509
4th	−14.47 ± 2.92	−4.95	<0.001 *	−11.93 ± 7.26	−1.64	0.107	−7.32 ± 3.30	−2.22	0.031 *	−35.06 ± 7.70	−4.56	<0.001 *	−0.02 ± 0.01	−1.72	0.091
5th	−15.80 ± 3.27	−4.83	<0.001 *	−23.73 ± 8.12	−2.92	0.005 *	−8.77 ± 3.69	−2.37	0.021 *	−8.95 ± 8.61	−1.04	0.304	−0.04 ± 0.01	−2.51	0.015 *
**Variables fitted as random effects**															
Horse	101.02 ± 53.63	1.88	0.060	2131 ± 1083	1.97	0.049	91.60 ± 50.12	1.83	0.068	732.08 ± 388.15	1.89	0.059	0.0005 ± 0.0003	1.54	0.124

^a^ regression coefficient (±SE) for variables fitted as fixed effects and variance (±SE) for variables fitted as random effects; ^b^ reference category; * significant at α = 0.05; abbreviations: IL-1β, interleukin 1β; IL-6, interleukin 6; IL-17, interleukin 17; TNF-α, tumour necrosis factor α; mRNA TNF-α, mRNA tumour necrosis factor α.

## Supplementary Materials

The following are available online at https://www.mdpi.com/2076-2615/9/9/616/s1, Table S1: Cytokines whose concentration was not significantly affected by repeated training in the mixed linear model; Table S2: Cytokine transcripts whose concentration was not significantly affected by repeated training in the mixed linear model.

## Abbreviations

APPs	Acute phase proteins
APR	Acute phase response
AST	Aspartate aminotransferase
β-Gus	β-glucuronidase
BMI	Body mass index
CPK	Creatine phosphokinase
CRP	C-reactive protein
HGB	Haemoglobin concentration
INFγ	Interferon γ
IL-1β	Interleukin 1β
IL-2	Interleukin 2
IL-4	Interleukin 4
IL-6	Interleukin 6
IL-10	Interleukin 10
IL-17	Interleukin 17
IL-1ra	Interleukin 1 receptor antagonist
K2-EDTA	K2-ethylenediaminetetraacetic acid
LPS	Lipopolysaccharide
miRNA	microRNA
PCV	Packed cell volume
PLT	Platelet count
WBC	White blood cell count
RBC	Red blood cell count
SAA	Serum amyloid A
sTNF-r1	Soluble tumour necrosis factor receptor 1
sTNF-r2	Soluble tumour necrosis factor receptor 2
TNF-α	Tumour necrosis factor α
TP	Total protein concentration
