# Supplementary material for: Anti-Inflammatory State in Arabian Horses Introduced to the Endurance Training
Source: Animals (Basel). 2019 Aug 27;9(9):616. doi: 10.3390/ani9090616 (PMC6769738; doi:10.3390/ani9090616)
Supplement: Supplementary file 1 [file animals-09-00616-s001.pdf]

Table S1 Cytokines whose concentration was not significantly affected by repeated training in the mixed linear model

	IL-2			IL-4			IL-10			INFγ		
Variable	Estimate of the model [a]	Parameter statistics	p-value	Estimate of the model [a]	Parameter statistics	p-value	Estimate of the model [a]	Parameter statistics	p-value	Estimate of the model [a]	Parameter statistics	p-value
Intercept	26.30±2.49	-	-	37.05±2.93	-	-	237.9±18.49	-	-	41.58±2.97	-	-
Variables fitted as fixed effects												
Time of blood collection												
before[b]	0	-	-	0	-	-	0	-	-	0	-	-
after	-1.41±1.48	-0.95	0.346	-0.84±1.46	-0.58	0.567	-12.92±7.37	-1.75	0.086	-2.14±1.37	-1.56	0.126
Training:												
1st[b]	0	-	-	0	-	-	0	-	-	0	-	-
2nd	-1.52±1.98	-0.77	0.447	-2.61±1.94	-1.34	0.185	2.50±9.83	0.25	0.801	-0.62±1.83	-0.34	0.737
3rd	4.08±2.17	1.88	0.066	-4.36±2.13	-2.05	0.046	5.75±10.81	0.53	0.597	-1.38±2.01	-0.68	0.497
4th	0.50±2.67	0.19	0.851	-5.25±2.63	-2.00	0.051	7.35±13.34	0.55	0.584	-5.15±2.49	-2.07	0.043
5th	0.12±2.98	0.04	0.969	-2.58±2.94	-0.88	0.385	-25.69±14.93	-1.72	0.092	-2.25±2.78	-0.81	0.423
Variables fitted as random effects												
Horse	33.12±19.63	1.69	0.092	55.45±30.32	1.83	0.067	2519±1328	1.90	0.058	60.07±32.32	1.86	0.063

[a] regression coefficient (±SE) for variables fitted as fixed effects and variance (±SE) for variables fitted as random effects; [b] reference category;); **Abbreviations**: IL-2, interleukin 2; IL-4, interleukin 4; IL-10, interleukin 10; INFγ, Interferon γ;

Table S2. Cytokine transcripts whose concentration was not significantly affected by repeated training in the mixed linear model

	**mRNA IL-1β**			**mRNA IL-6**			**mRNA IL-10**		
Variable	Estimate of the model [a]	Parameter statistics	p-value	Estimate of the model [a]	Parameter statistics	p-value	Estimate of the model [a]	Parameter statistics	p-value
Intercept	0.10 ±0.05	-	-	-0.003 ±0.007	-	-	0.002 ±0.001	-	-
Variables fitted as fixed effects									
Time of blood collection									
before[b]	0	-	-	0	-	-	0	-	-
after	0.11 ±0.04	2.77	0.008	-0.005 ±0.006	-0.89	0.380	-0.001 ±0.001	-0.65	0.518
Training:									
1st[b]	0	-	-	0	-	-	0	-	-
2nd	0.06 ±0.05	1.25	0.217	-0.000 ±0.008	-0.02	0.988	0.001 ±0.002	0.36	0.721
3rd	0.03 ±0.06	0.45	0.651	-0.000 ±0.009	-0.01	0.989	0.003 ±0.002	1.48	0.144
4th	-0.02 ±0.07	-0.24	0.808	-0.000 ±0.011	-0.04	0.972	0.001 ±0.002	0.26	0.799
5th	0.08 ±0.07	1.04	0.301	0.074 ±0.012	6.23	<0.001	0.004 ±0.002	2.00	0.050
Variables fitted as random effects									
Horse	0.01 ±0.01	1.19	0.234	0.000 ±0.000	0.33	0.744	0	0	0

[a] regression coefficient (±SE) for variables fitted as fixed effects and variance (±SE) for variables fitted as random effects; [b] reference category;); **Abbreviations**: mRNA IL-1β, mRNA interleukin 1β; mRNA IL-6, mRNA interleukin 6; mRNA IL-10, mRNA interleukin 10;
